# Supplementary material for: Identification of photoperiod-induced specific miRNAs in the adrenal glands of Sunite sheep (Ovis aries)
Source: Front Vet Sci. 2022 Jul 22;9:888207. doi: 10.3389/fvets.2022.888207 (PMC9354845; doi:10.3389/fvets.2022.888207)
Supplement: Supplementary file 1 [file Data_Sheet_1.ZIP › Supplementary file/miRDeep2 file.zip!/miRDeep2 file/Know_Structure/SPLP42A3/oar-mir-148a.pdf]

miRBase precursor : oar-mir-148a
Total read count : 6584029
oar-miR-148a read count : 6579083
remaining reads : 4946

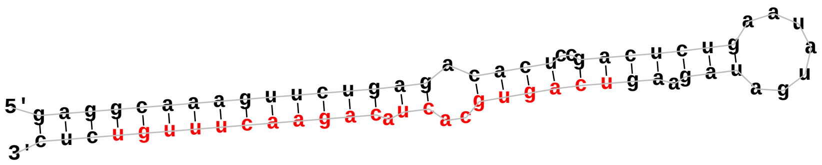

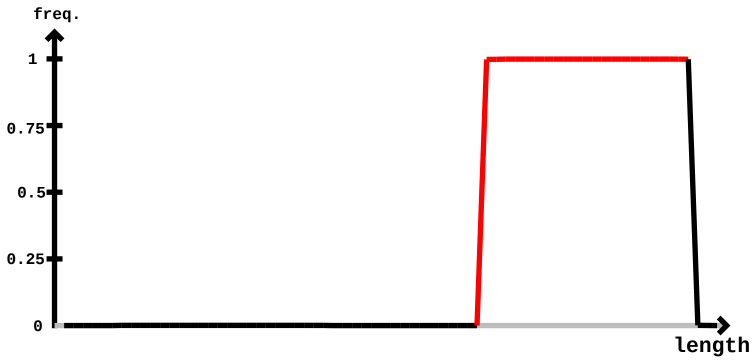

oar-miR-148a

```
5'- gaggcaaaguucugagacacuccgacucugaauaugauagaagucagugcacuacagaacuuugucuc  -3'   exp
    ((((((((((((((((.((((..(((((((.......))).))))))))..)).))))))))))))))   reads   mm   sample
    ...gcaaaguucugagacacuccgacu.........................................   1       0    seq
    ....caaaguucugagacacuccga...........................................   2       0    seq
    ....caaaguucugagacacuccgac..........................................   2       0    seq
    ....caaaguucugagacacuccgacu.........................................   103     0    seq
    .....aaaguucugagacacuccg............................................   5       0    seq
    .....aaaguucugagacacuccga...........................................   44      0    seq
    .....aaaguucugagacacuccgac..........................................   40      0    seq
    .....aaaguucugagacacuccgacu.........................................   4733    0    seq
    .....aaaguucugagacacuccgacuc........................................   11      0    seq
    .....aaaguucugagacacuccgacucug......................................   1       0    seq
    ......aaguucugagacacuccgacu.........................................   2       0    seq
    .....................................uagaagucagugcacuacagaacuuugu...   1       0    seq
    ......................................agaagucagugcacuacagaacuuugu...   1       0    seq
    ..........................................guUagugcacuacagaacuuugu...   1       1    seq
    ..........................................gucagugcacuacagaacuuugu...   165     0    seq
    ...........................................ucagugcacuacagaac........   6       0    seq
    ...........................................ucaCugcacuacagaacu.......   3       1    seq
    ...........................................ucagugcacuacagaacu.......   114     0    seq
    ...........................................uUagugcacuacagaacu.......   1       1    seq
    ...........................................ucagugcacuacagaacuu......   271     0    seq
    ...........................................ucagugcacuacagaacuuu.....   1108    0    seq
    ...........................................uUagugcacuacagaacuuu.....   3       1    seq
    ...........................................ucagugcacuacagaacuuug....   2625    0    seq
    ...........................................uUagugcacuacagaacuuug....   5       1    seq
    ...........................................uUagugcacuacagaacuuugu...   9381    1    seq
    ...........................................ucagugcacuacagaacuuugu...   6549418 0    seq
    ...........................................uUagugcacuacagaacuuuguc..   8       1    seq
    ...........................................ucagugcacuacagaacuuuguc..   4245    0    seq
    ...........................................ucagugcacuacagaacuuugucu.   2564    0    seq
    ...........................................ucagugcacuacagaacuuugucuc   10      0    seq
    ............................................Uagugcacuacagaacuuugu...   29      1    seq
    ............................................cagugcacuacagaacuuugu...   3602    0    seq
    ............................................cagugcacuacagaacuuuguc..   7       0    seq
    ............................................cagugcacuacagaacuuugucu.   1       0    seq
    .............................................agugcacuacagaacuuug....   1       0    seq
```

oar-miR-148a

```
gaggcaaaguucugagacacuccgacucugaauaugauagaagucagugcacuacagaacuuugucuc
.............................................agugcacuacagaacuuugu...   5437   0   seq
.............................................agugcacuacagaacuuuguc..   5      0   seq
.............................................agugcacuacagaacuuugucu.   1      0   seq
..............................................gugcacuacagaacuuugu...   17     0   seq
...............................................ugcacuacagaacuuugu...   9      0   seq
................................................gcacuacagaacuuugA...   2      1   seq
................................................gcacuacagaacuuugu...   43     0   seq
................................................gcacCacagaacuuugu...   1      1   seq
```
